# Inverse Association Between Betel Quid Use and Diabetes in Rural Bangladesh

**DOI:** 10.1002/ajhb.70203

**Published:** 2026-02-13

**Authors:** Kristin K. Sznajder, Mary K. Shenk, Laura Perez, Nurul Alam, Rubhana Raqib, Anjan Kumar, Farjana Haque, Tami Blumenfield, Siobhán M. Cully, Katherine Wander

**Affiliations:** ^1^ Pennsylvania State University, College of Medicine Department of Public Health Sciences Hershey Pennsylvania USA; ^2^ Pennsylvania State University Department of Anthropology Hershey Pennsylvania USA; ^3^ Icddr,b Dhaka Bangladesh; ^4^ University of New Mexico Department of Anthropology Albuquerque New Mexico USA; ^5^ Department of Anthropology Rutgers University New Brunswick New Jersey USA; ^6^ Binghamton University, State University of New York Department of Anthropology Binghamton New York USA

**Keywords:** areca nut, Bangladesh, betel nut, evolutionary medicine, insulin resistance, substance use

## Abstract

**Objectives:**

Betel quid is used as a mild stimulant in many parts of South and East Asia and the Pacific. In observational studies, its use has been associated with elevated risk for diabetes, but studies in animal models suggest some component(s) of betel quid could reduce risk.

**Methods:**

We assessed associations between betel quid use and diabetes (glycated hemoglobin, HbA_1c_ ≥ 6.5%) among a cross‐sectional sample of 410 men and 717 non‐pregnant women in Matlab, Bangladesh.

**Results:**

In multivariable logistic regression, betel quid use was inversely associated with diabetes among men (aOR: 0.45; 95% CI: 0.26, 0.79) but not women (aOR: 0.88; 95% CI: 0.51, 1.52). There was a dose–response relationship between frequency of betel quid use and diabetes among men (aOR: 0.73; 95% CI: 0.59, 0.89), but not women (aOR: 0.96; 95% CI: 0.77, 1.18). Betel quid use was inversely associated with diabetes as an ordinal variable (no diabetes/prediabetes/diabetes) among men (aOR: 0.55; 95% CI: 0.36, 0.82) and women (aOR: 0.66; 95% CI: 0.47, 0.94). Structural equation modeling suggested that some of the inverse association was mediated by food source (market vs. household production).

**Conclusions:**

These models support the hypothesis that betel quid use could decrease, rather than increase, risk for diabetes in the Bangladeshi context, particularly among men (who have more frequent betel quid use than women). Heterogeneity in betel quid preparation across settings, multifactorial effects of betel quid use, and publication bias may contribute to differences between these findings and other observational studies.

## 1 Introduction

Betel (or areca) nut is a psychoactive substance (Murphy and Herzog 2015) used by ~10% of the world's population, primarily in South and Southeast Asia and Oceania (Heck et al. 2012; International Agency for Research on Cancer 2012; Lee et al. 2011). Preparation of the betel quid varies across contexts, but usually includes the betel nut and calcium hydroxide (slaked lime), often mixed with tobacco, wrapped in a betel leaf (which refers to a climbing vine, distinct from the palm tree from which betel nut is derived), often with spices (e.g., cardamom, clove) or other ingredients included for taste (IARC Working Group on the Evaluation of Carcinogenic Risks to Humans 2004). This quid is then chewed or held between the teeth and the cheek, allowing mucous membranes of the mouth to absorb the psychoactive ingredient in areca nut, arecoline.

Like many psychoactive substances used in traditional medicine, betel quid seems to have mixed effects on health (summarized in Supporting Information Table S1). Consumers have reported an improved sense of well‐being, increased energy, and better digestion (Boucher and Mannan 2002). Research with schizophrenia patients found milder symptoms in those who chewed betel quid (Blount et al. 2002; Sullivan et al. 2000; Sullivan et al. 2007). Betel leaf and its component chemicals have been associated with beneficial health effects in humans and animal models, including anticarcinogenic effects, increased antioxidant activity, and antibacterial and antihelminthic properties (Chakraborty and Shah 2011; Datta et al. 2011; Padma et al. 1989; Toprani and Patel 2013). By contrast, betel quid use has been associated with periodontal disease and damage to teeth, as well as increased risk for cardiovascular disease, hypertension, and oropharyngeal cancer (Anand et al. 2014; Chang et al. 2006; Lin et al. 2008; Tseng 2008). Studies have also found a positive association between betel quid consumption and type 2 diabetes (T2D), and poorer glycemic control (a component of T2D disease etiology) among betel quid users in Taiwan and London (Benjamin 2001; Mannan et al. 2000; Tseng 2010; Tung et al. 2004).

The link between betel quid consumption and T2D is of particular interest because epidemiologic findings among humans seem to contradict laboratory studies, many of which indicate potential antidiabetic effects of betel quid ingredients in induced animal models (Ahmed et al. 2022; Amudhan and Begum 2008; Arambewela et al. 2005; Huang et al. 2013; Musdja et al. 2020; Santhakumari et al. 2006). The mechanism(s) by which betel quid use may affect glycemic control and diabetes are summarized in Table 1. Alkaloids from betel quid may inhibit gamma‐aminobutyric acid (GABA) receptors, which normally inhibit glucagon secretion (Boucher and Mannan 2002), increasing blood glucagon and ultimately blood glucose (Lin et al. 2008). Other studies propose direct toxicity of one or multiple betel quid components to pancreatic beta cells (which secrete insulin) (Boucher et al. 1994). An increased risk of obesity associated with betel quid consumption has also been proposed as a potential contributor to the development of T2D in these populations (Lin et al. 2009; Mannan et al. 2000). Yet, betel quid consumption has also been associated with appetite suppression (Strickland and Duffield 1997; Strickland et al. 2003), which some studies have found to be associated with lower body mass index (BMI) (Suzuki et al. 2012).

**TABLE 1:** Laboratory and epidemiologic studies of betel quid and diabetes.

Author and Year	Subject	T2D risk direction	Proposed mechanism
**Laboratory studies**			
Ahmed et al. 2022	In silico—molecular docking study	Decreased	Reduced blood glucose via inhibition of alpha‐amylase and alpha‐glucosidase, enzymes that support intestinal breakdown and absorption of glucose
Amudhan and Begum 2008	Animal—rats	Decreased	Inhibited intestinal absorption of monosaccharides resulting in reduced postprandial blood glucose levels
Arambewela et al. 2005	Animal—rats	Decreased	Increased storage of glycogen in liver and muscles through “insulinomimetic” effects
Cardosa et al. 2021	Human cell line—transcriptomics	Increased	Increased expression of diabetes‐ and obesity‐associated genes
Hsieh et al. 2011	Murine cell line	Increased	Decrease insulin signaling in adipocytes. Disrupted phosphorylation and function of downstream molecules in the insulin signaling cascade, leading to impaired insulin signaling in adipocytes
Huang et al. 2013	Animal—mice	Decreased	Reduced gluconeogenesis due to reduced expression of key enzymes, phosphoenol‐pyruvate carboxykinase and glucose‐6‐phosphatase
Musdja et al. 2020	Animal—rats	Decreased	Lowered blood glucose and other anti‐diabetic effects of alkaloids (e.g., arecoline)
Owen et al. 2008	Murine cell line	Increased	Disrupted insulin‐mediated glucose intake in adipocytes
Santhakumari et al. 2006	Animal—rats	Decreased	Decreased liver fructose‐1,6‐bisphosphatase and glucose‐6‐phosphatase activity (which support gluconeogenesis) and increased hexokinase activity promoting glycolysis or glycogen synthesis (betel leaf, rather than betel nut, components)
**Epidemiologic studies**			
Benjamin 2001	Humans (Papua New Guinea)	Increased	A positive association was found between betel nut chewing and high fasting capillary blood glucose.
Lin et al. 2008	Humans (Taiwan)	No association	No association identified between betel quid and diabetes among men; betel quid chewers had higher BMI and waist circumference than never chewers
Mannan et al. 2000	Humans (London, Bangladeshi migrants)	Increased	Betel use interacted with sex to increase glycemia in females only; increased central obesity
Tseng 2010	Human (Taiwan)	Increased	None discussed
Tung et al. 2004	Human (Taiwan)	Increased	Direct toxicity to pancreatic beta cells

Betel quid use varies widely across Asia and Oceania, along multiple dimensions, including preparation of the betel nut itself, preparation of the betel quid, and use patterns, so it is not reasonable to expect associations between betel quid use and T2D to be consistent across populations. Indeed, geographic heterogeneity in associations between betel quid use and T2D may help to explain the apparent contrast between observational epidemiologic studies and laboratory‐based studies. Thus, we assessed associations between betel quid consumption and diabetes among a cross‐sectional sample of men and women living in Matlab, Bangladesh. We further assessed pathways (e.g., BMI) hypothesized to connect betel quid use and T2D.

## 2 Materials and Methods

### 2.1 Research Setting

We used data collected as part of a larger study on demographic indicators of health and fertility conducted in Matlab, Bangladesh, a densely populated rural area undergoing rapid market integration. Both men and women in this area of Bangladesh commonly chew betel quid, though men chew it more frequently than women, and people from lower socioeconomic status groups use betel quid at higher rates (Wu et al. 2015). Matlab is undergoing a nutrition transition with high and increasing rates of diabetes (Khan et al. 2013; Khan and Talukder 2013; Sznajder et al. 2021).

### 2.2 Study Design and Participants

A random, age‐stratified sample of women was drawn from a population roster of over 230 000 people in the region maintained by the Matlab Health and Demographic Surveillance System (HDSS), which has operated since the late 1960s by icddr,b (formerly the International Centre for Diarrhoeal Disease Research, Bangladesh) (Alam et al. 2017). Selected women were eligible to participate if they were between 20 and 65 years of age during the first data collection wave in 2010.

Men were eligible to participate if they were married to a participating woman and were present in Matlab at the time of the second data collection wave in 2017; many husbands were not present due to high rates of male labor migration to cities in Bangladesh or abroad (Sznajder et al. 2021). This approach ensured a sample that is representative of adult residents of the study area of Matlab, Bangladesh, which for men is disproportionately older and thus potentially in poorer health than a nationally representative sample due to labor migration (Alam et al. 2024). The gender distribution of our sample accurately reflects the actual gender distribution of the population of Matlab (icddr,b 2020), as well as many other rural areas of Bangladesh, given the very high rates of male labor outmigration in the county.

All survey, anthropometric, and biomarker data analyzed here were collected in 2017–2018 from 765 women (98% of the 2010 participants who were still alive and continued to reside in the study area), and 499 of their husbands (excluding husbands who were deceased or working as labor migrants outside of Matlab). We excluded participants who reported being pregnant at the time of data collection (*N* = 4), for whom we did not have HbA_1c_ test results (*N* = 114), or for whom we were missing other pertinent variables (*N* = 19) from these analyses.

### 2.3 Measurements

Data collection instruments were developed and piloted by Shenk and Alam and then implemented by field staff trained at icddr,b. Betel quid use was assessed via survey items that asked participants if they chewed betel quid (yes or no). If yes, participants were asked to report their typical frequency of use, either as times per month or times per day. Based on the reported typical frequency, betel quid use was categorized as none, used monthly, used daily but less than five times per day, and used at least five times per day. Additional variables characterized via surveys included age, years of education, the MacArthur Scale of Subjective Social Status (Adler and Stewart 2007), occupation (for men only, dichotomized for these analyses as manual laborer or not), social network size (the number of people who the participant interacted with regularly across various social domains including childcare/work assistance, loans of goods or money, and different types of social and emotional support (Shenk et al. 2021)), food security (dichotomized for these analyses as always having enough quality food compared with not always having enough quality food), and food source (all food purchased from the bazaar, where both whole and processed foods are available, compared with some or no food purchased from the bazaar, meaning that food was primarily sourced from the family's own subsistence activities).

Anthropometry was characterized after survey completion. A stadiometer (Seca 213) was used to measure height in centimeters and a digital scale (Tanita bc 545) was used to measure weight in kilograms (kgs) (Wang and Hui 2015). BMI was calculated as weight (in kgs) divided by height (in meters) squared and categorized as obese (≥ 27.5), overweight (23–< 27.5), lean (18.5–< 23), or underweight (< 18.5) according to the World Health Organization's recommendations for Asian populations (Weir and Jan 2019). Grip strength was estimated in kgs with a digital hand dynamometer (Takei Grip‐D, TKK 5401). Three trials on each hand were included; for these analyses, we used the first trial for the right hand.

A small amount of capillary whole blood was collected from participants by finger stick and immediately evaluated for glycated hemoglobin (HbA_1c_; A1C Now, PTS Diagnostics (Hirst et al. 2017)). Additional drops of whole blood were collected on filter paper (Whatman #903) for dried blood spots (DBS) and allowed to dry for up to 24 h. DBS were then frozen at −20°C until analysis.

We used 5.7% ≤ HbA_1c_ < 6.5% to capture prediabetes and HbA_1c_ ≥ 6.5% to capture diabetes and used both binary (no diabetes, diabetes) and ordinal (no diabetes, prediabetes, diabetes) variables to describe diabetes. C‐reactive protein (CRP), a biomarker of inflammation, was estimated in DBS at the icddr,b Immunobiology, Nutrition, and Toxicology Laboratory using an enzyme immunoassay kit (BioCheck BC‐1119). Kit instructions were modified for DBS as follows: one 3.2 mm disc of DBS specimen was isolated by a manual punch and eluted overnight in assay buffer at 4°C. Eluent was assayed without further dilution. Dilution correction was performed assuming 1.5 μL serum equivalent in a 3.2 mm disc (Brindle et al. 2010). Across 37 plates, the intra‐assay coefficient of variation was 5.7% and the inter‐assay coefficient of variation was 16.3% (16.7% for a second control specimen included on 27 of these plates). Elevated CRP was defined as > 3 mg/L and < 10 mg/L; participants with CRP ≥ 10 mg/L were excluded from models that considered the CRP variable, as concentrations in this range may reflect infectious disease processes rather than an individual's characteristic background concentration (Mac Giollabhui et al. 2020; Wander et al. 2012).

### 2.4 Statistical Analysis

We estimated logistic regression models of associations between betel quid use and diabetes, and ordinal logistic regression models of associations between betel quid and the ordinal variable of no diabetes, prediabetes, and diabetes. We first estimated crude odds ratios (cOR). We then estimated adjusted odds ratios (aOR) controlling for potentially confounding variables that are likely causally upstream of both betel quid use and diabetes: age, education, MacArthur Scale (for socioeconomic status), and occupation (for men only; most women identified their occupation as housewife).

We further assessed potential mediators—variables that are conceivably downstream of the physiological, behavioral, or social effects of betel quid use—using structural equation models (SEM). Mediation analysis allowed us to assess hypotheses that betel quid use acted through non‐physiologic means, by altering participants' social network size, reliance on purchased (vs. produced) food, or food security (or perception thereof) and through these variables, their T2D risk. All of these factors could impact T2D risk in complex ways, including through diets, access to medical care, and psychosocial stress, but do not suggest a direct physiologic impact of betel quid on diabetes etiology. Mediation analyses further allowed us to address some physiologic pathways by which betel quid could impact diabetes risk, including effects of betel quid use on BMI, CRP, and muscle mass or strength (grip strength). This expansive approach to assessing mediating variables allowed us to assess both direct physiologic effects of betel quid on diabetes risk and indirect effects via food source, food quantity, or social interactions, adding nuance to our assessment of causal inference from these models.

To evaluate the potential influence of residual confounding, we conducted a sensitivity analysis of our regression models predicting both dichotomous and ordinal diabetes outcomes using E‐values (Gaster et al. 2023; VanderWeele and Ding 2017). An E‐value quantifies the minimum strength of association that an unmeasured confounder would need to have with both the exposure and the outcome, on the risk ratio scale, to fully explain an observed association. Higher E‐values indicate that the observed association is more robust to potential unmeasured confounding. The square root of the odds ratios was taken in order to approximate the risk ratio for the E‐value calculation.

To assess the extent to which models were robust to under‐sampling of particularly healthy men due to labor migration, we evaluated logistic regression models restricted to age ≥ 55 years, as labor migration is quite rare after this age (Alam et al. 2017). All data were analyzed in SAS 9.4.

### 2.5 Research Ethics Approval

Procedures were approved by the Pennsylvania State University Institutional Review Board and the Ethical Review Committee at icddr,b and conformed to the ethical standards of the Declaration of Helsinki and the US Federal Policy for the Protection of Human Subjects. Participants were made aware that study participation was voluntary and provided written consent to participate. HbA_1c_ results (HbA_1c_ in %; diabetic/not), height, weight, BMI, and results of testing not analyzed here were shared with participants (and provided in writing) at the time of data collection.

## 3 Results

Data on diabetes were available for 1127 people, 410 men and 717 non‐pregnant women (Table 2). 175 (15.5%) participants were diabetic. Diabetes was more common among men (20.0%) than women (13.0%; *p* = 0.002). Betel quid use (any/none) was similar between men (52.7%) and women (53.4%; *p* = 0.812); however, more men (33.2%) than women (22.2%; *p* < 0.001) reported the highest category of betel quid use (at least five times a day). Participating men were older (56.2 ± 12.1 years) than women (49.9 ± 12.3 years; *p* < 0.001), had more years of education (4.9 ± 4.5 years) than women (4.1 ± 3.9 years; *p* = 0.002), had a greater grip strength (26.4 ± 10.7 kgs) than women (16.4 ± 4.6 kgs; *p* < 0.001), and reported larger social networks (12.7 ± 3.9 people) than women (10.4 ± 3.8 people; *p* < 0.001). Women reported higher socioeconomic status by MacArthur Ladder (4.6 ± 1.8) than men (3.9 ± 1.2; *p* < 0.001), were more likely to obtain all their food from the bazaar (63.1%) than men (47.7%; *p* < 0.001), and had higher BMI (23.5 ± 4.2 kg/m^2^) than men (21.5 ± 3.6 kg/m^2^; *p* < 0.001). These gender differences in socioeconomic status likely result from labor migration of younger men to cities in Bangladesh or abroad, including some who would otherwise have participated in our research. Differences in outcomes and key covariates by betel quid use and gender are further described in Supporting Information (Table S1).

**TABLE 2:** Sample characteristics *N* = 1127.

	*N* (column %)/Mean (SD)			
	Total	Men *N* = 410	Women *N* = 717	p*
Betel quid use	599 (53.1)	216 (52.7)	383 (53.4)	0.812
No betel quid use	528 (46.9)	194 (47.3)	334 (46.6)	
Betel quid use ≥ 5 times per day	294 (26.2)	135 (33.2)	159 (22.2)	< 0.001
Betel quid use < 5 times per day	268 (23.9)	72 (17.7)	196 (27.4)	
Betel quid use monthly	33 (2.9)	6 (1.5)	27 (3.8)	
No betel quid use	528 (47.0)	194 (47.7)	334 (46.7)	
Diabetes	175 (15.5)	82 (20.0)	93 (12.9)	< 0.001
Prediabetes	460 (40.8)	178 (43.4)	282 (39.3)	
No diabetes	492 (43.7)	150 (36.6)	342 (47.7)	
Age	52.2 (±12.6)	56.2 (±12.1)	49.9 (±12.3)	< 0.001
MacArthur Ladder	4.3 (±1.7)	3.9 (±1.2)	4.6 (±1.8)	< 0.001
Education	4.4 (±4.2)	4.9 (±4.5)	4.1 (±3.9)	0.002
No school	355 (31.8)	113 (27.8)	242 (33.9)	0.028
Up to primary	385 (34.4)	137 (33.7)	248 (34.8)	
More than primary	378 (33.8)	156 (38.4)	222 (31.2)	
Food secure	752 (67.0)	280 (68.6)	472 (66.1)	0.388
Not food secure	370 (32.9)	128 (31.4)	242 (33.9)	
Food from the bazaar	646 (57.5)	194 (47.7)	452 (63.1)	< 0.001
Not all food from bazaar	477 (42.5)	213 (52.3)	264 (36.9)	
Men who are laborers	218 (54.4)	218 (54.4)		
Men of other occupation	183 (45.6)	183 (45.6)		
BMI	22.8 (±4.1)	21.5 (±3.6)	23.5 (±4.2)	< 0.001
Obese ≥ 27.5	150 (13.4)	29 (7.1)	121 (16.9)	< 0.001
Overweight 23–< 27.5	345 (30.7)	100 (24.5)	245 (34.3)	
Lean 18.5–< 23	460 (40.9)	188 (45.9)	272 (38.0)	
Underweight< 18.5	169 (15.0)	92 (22.5)	77 (10.8)	
CRP > 3– < 10	240 (22.1)	78 (19.8)	162 (23.4)	0.161
CRP ≤ 3	847 (77.9)	317 (80.3)	530 (76.6)	
Grip strength (R1)	19.9 (±7.4)	26.1 (±7.5)	16.5 (±4.5)	< 0.001
Social network size	11.2 (±4.0)	12.7 (±3.9)	10.4 (±3.8)	< 0.001

*Note:* *Chi‐square test for gender difference in means.

Betel quid use and gender interacted in their association with diabetes (*p* < 0.01; Table S5), so all further regression and mediation analyses were stratified by gender. Stratified crude models show an inverse association between betel quid use and diabetes among men (cOR: 0.50; 95% CI: 0.31, 0.82, Table 3, Panel 1) and a positive association among women (cOR: 1.61; 95% CI: 1.02, 2.52, Table 4, Panel 1).

**TABLE 3:** Crude and adjusted logistic regression models of the association between betel quid use and diabetes among men.

	Logistic regression (HbA_1c_ ≥ 6.5%)	Logistic regression (HbA_1c_ ≥ 6.5%)	Ordinal logistic regression (HbA_1c_ < 5.6%; 5.7% ≤ HbA_1c_ < 6.5; HbA_1c_ ≥ 6.5%)	Ordinal logistic regression (HbA_1c_ < 5.6%; 5.7% ≤ HbA_1c_ < 6.5; HbA_1c_ ≥ 6.5%)
**Panel 1 (Crude)**	**OR (CI)**	**OR (CI)**	**OR (CI)**	**OR (CI)**
Betel quid use	0.50 (0.31, 0.82)		0.65 (0.45, 0.93)	
Betel quid use ≥ 5/day		0.47 (0.26, 0.84)		0.61 (0.40, 0.92)
Betel quid use < 5/day		0.41 (0.19, 0.89)		0.68 (0.41, 1.13)
Betel quid use monthly		2.88 (0.56, 14.73)		2.04 (0.44, 9.33)
No betel quid use		REF		REF
**Panel 2 (Adjusted for confounders)**	**OR (CI)**	**OR (CI)**	**OR (CI)**	**OR (CI)**
Betel quid use	0.45 (0.26, 0.79)		0.55 (0.36, 0.82)	
Betel quid use ≥ 5/day		0.40 (0.21, 0.76)		0.48 (0.30, 0.75)
Betel quid use < 5/day		0.36 (0.16, 0.83)		0.56 (0.32, 0.97)
Betel quid use monthly		4.36 (0.59, 32.04)		3.69 (0.63, 21.64)
No betel quid use		REF		REF
Age	1.01 (0.99, 1.04)	1.02 (0.99, 1.04)	1.02 (0.99, 1.03)	1.02 (1.00, 1.04)
MacArthur Ladder	1.33 (1.06, 1.68)	1.31 (1.04, 1.66)	1.21 (1.02, 1.44)	1.19 (1.00, 1.42)
Education	0.99 (0.93, 1.06)	0.99 (0.93, 1.05)	0.99 (0.95, 1.04)	0.99 (0.94, 1.04)
Laborer	0.48 (0.27, 0.85)	0.49 (0.28, 0.88)	0.69 (0.45, 1.05)	0.71 (0.46, 1.07)
**Panel 3 (Adjusted for confounders and mediators)**	**OR (CI)**	**OR (CI)**	**OR (CI)**	**OR (CI)**
Betel quid use	0.46 (0.25, 0.86)		0.62 (0.41, 0.96)	
Betel quid use ≥ 5/day		0.39 (0.19, 0.81)		0.53 (0.33, 0.86)
Betel quid use < 5/day		0.39 (0.16, 0.96)		0.63 (0.35, 1.13)
Betel quid use monthly		12.08 (0.61, 217.64)		11.74 (0.95, 145.14)
No Betel quid use		REF		REF
Age	0.99 (0.97, 1.03)	1.00 (0.97, 1.04)	1.01 (0.99, 1.03)	1.01 (0.99, 1.03)
MacArthur Ladder	1.42 (1.07, 1.89)	1.37 (1.03, 1.82)	1.20 (0.99, 1.47)	1.17 (0.96, 1.42)
Education	0.95 (0.88, 1.03)	0.95 (0.88, 1.02)	0.98 (0.93, 1.03)	0.97 (0.92, 1.03)
Laborer	0.75 (0.38, 1.49)	0.78 (0.39, 1.56)	1.00 (0.62, 1.62)	1.02 (0.63, 1.66)
Food security	1.51 (0.75, 3.05)	1.48 (0.73, 3.01)	1.26 (0.80, 1.99)	1.24 (0.78, 1.95)
Food from the bazaar	1.98 (1.05, 3.74)	1.93 (1.02, 3.67)	1.69 (1.09, 2.62)	1.64 (1.06, 2.55)
BMI	1.19 (1.09, 1.29)	1.19 (1.09, 1.29)	1.13 (1.06, 1.20)	1.14 (1.07, 1.21)
CRP > 3 < 10	0.80 (0.38, 1.66)	0.83 (0.39, 1.74)	0.91 (0.55, 1.51)	0.91 (0.55, 1.52)
Grip strength	0.95 (0.91, 0.99)	0.95 (0.90, 0.99)	0.97 (0.94, 1.01)	0.97 (0.94, 1.00)
Social network size	0.99 (0.93, 1.07)	1.01 (0.94, 1.09)	0.99 (0.94, 1.04)	0.99 (0.94, 1.05)

**TABLE 4:** Crude and adjusted logistic regression models of the association between betel quid use and diabetes among women.

	Logistic regression (HbA_1c_ ≥ 6.5%)	Logistic regression (HbA_1c_ ≥ 6.5)	Ordinal logistic regression (HbA_1c_ < 5.6%; 5.7% ≤ HbA_1c_ < 6.5; HbA_1c_ ≥ 6.5%)	Ordinal logistic regression (HbA^1c^ < 5.6%; 5.7% ≤ HbA_1c_ < 6.5; HbA_1c_ ≥ 6.5%)
**Panel 1 (Crude)**	**OR (CI)**	**OR (CI)**	**OR (CI)**	**OR (CI)**
Betelnut Use	1.61 (1.02, 2.52)		1.24 (0.94, 1.64)	
Betel quid use ≥ 5/day		1.49 (0.85, 2.63)		1.19 (0.84, 1.72)
Betel quid use < 5/day		1.79 (1.07, 2.99)		1.36 (0.98, 1.91)
Betel quid use monthly		1.10 (0.32, 3.86)		0.73 (0.34, 1.59)
No Betel quid use		REF		REF
**Panel 2 (Adjusted for confounders)**	**OR (CI)**	**OR (CI)**	**OR (CI)**	**OR (CI)**
Betelnut use	0.88 (0.51, 1.52)		0.66 (0.47, 0.94)	
Betel quid use ≥ 5/day		0.86 (0.45, 1.65)		0.67 (0.44, 1.02)
Betel quid use < 5/day		0.91 (0.49, 1.68)		0.69 (0.46, 1.04)
Betel quid use monthly		0.76 (0.21, 2.75)		0.49 (0.22, 1.09)
No Betel quid use		REF		REF
Age	1.05 (1.02, 1.07)	1.05 (1.02, 1.07)	1.04 (1.03, 1.06)	1.04 (1.03, 1.06)
MacArthur Ladder	1.12 (0.99, 1.27)	1.12 (0.99, 1.27)	1.06 (0.98, 1.15)	1.06 (0.98, 1.15)
Education	0.99 (0.92, 1.07)	0.99 (0.92, 1.07)	0.98 (0.94, 1.03)	0.98 (0.94, 1.03)
**Panel 3: (Adjusted for confounders and mediators)**	**OR (CI)**	**OR (CI)**	**OR (CI)**	**OR (CI)**
Betelnut Use	1.03 (0.56, 1.88)		0.74 (0.51, 1.07)	
Betel quid use ≥ 5/day		0.66 (0.17, 2.55)		0.71 (0.46, 1.12)
Betel quid use < 5/day		1.17 (0.59, 2.30)		0.85 (0.55, 1.29)
Betel quid use monthly		0.97 (0.48, 1.97)		0.44 (0.19, 1.01)
No Betel quid use		REF		REF
Age	1.04 (1.01, 1.07)	1.04 (1.01, 1.07)	1.04 (1.02, 1.06)	1.04 (1.02, 1.06)
MacArthur Ladder	1.06 (0.93, 1.22)	1.05 (0.91, 1.21)	1.02 (0.93, 1.12)	1.01 (0.93, 1.11)
Education	0.94 (0.86, 1.02)	0.94 (0.86, 1.02)	0.94 (0.89, 0.99)	0.94 (0.89, 0.99)
Food security	1.30 (0.73, 2.32)	1.31 (0.73, 2.34)	1.07 (0.76, 1.51)	1.09 (0.77, 1.54)
Food from the bazaar	1.01 (0.59, 1.71)	0.99 (0.59, 1.69)	1.21 (0.88, 1.68)	1.23 (0.89, 1.69)
BMI	1.09 (1.03, 1.17)	1.09 (1.03, 1.17)	1.10 (1.06, 1.15)	1.11 (1.06, 1.15)
CRP > 3‐ < 10	2.23 (1.31, 3.77)	2.27 (1.34, 3.86)	1.59 (1.11, 2.30)	1.62 (1.12, 2.34)
Grip strength (R1)	0.97 (0.92, 1.03)	0.97 (0.92, 1.03)	0.99 (0.96, 1.03)	0.99 (0.96, 1.03)
Social Network Size	1.05 (0.99, 1.12)	1.06 (0.99, 1.12)	1.03 (0.99, 1.08)	1.04 (0.99, 1.08)

In multivariable models, betel quid use was independently inversely associated with diabetes among men (aOR: 0.45; 95% CI: 0.26, 0.79; Table 3, Panel 2). Further, in ordinal logistic regression using the categorical outcome no diabetes/prediabetes/diabetes, betel quid use was independently inversely associated with diabetes progression among men (aOR: 0.55; 95% CI: 0.36, 0.82; Table 3, Panel 2). E‐values for associations between betel quid use and dichotomous diabetes (2.24; 95% CI: 1.31, 3.33) and the ordinal diabetes outcome (1.86; 95% CI: 1.19, 2.55) among men suggest that an uncontrolled confounding variable would need to have a moderate to strong association with both betel quid use and diabetes to fully explain the observed associations. Finally, a test for trend to examine a dose–response relationship between the frequency of betel quid use and diabetes supported the finding of an inverse association between diabetes and betel quid use among men (aOR: 0.73; 95% CI: 0.59, 0.89), considering the same variables in Table 3, Panel 2.

Models restricted to men age ≥ 55 years (who are much less likely to be migrants) were similar: adjusted for the confounders in Table 3, Panel 2, betel quid use was inversely associated with diabetes (aOR: 0.36; 95% CI: 0.18, 0.71) (Table S6).

Among women, betel quid use (any/none) was not associated with diabetes (aOR: 0.88; 95% CI: 0.51, 1.52; Table 4, Panel 2) in multivariable analyses. However, betel quid use was inversely associated with diabetes disease progression among women in ordinal logistic regression (aOR: 0.66; 95% CI: 0.47, 0.94; Table 4, Panel 2). The test for trend (controlling for the same confounding variables) did not reveal an association between diabetes and increasing frequency of betel quid use (aOR: 0.96; 95% CI: 0.77, 1.18).

Proportional odds assumptions were met for all ordinal logistic regression models, meaning the logit surfaces were parallel and the OR were constant across outcome categories.

SEM revealed only one potential causal pathway among the mediating variables we considered among men: betel quid use was inversely associated with food from the bazaar (coef: −0.15; *p*: 0.003) and food from the bazaar was positively associated with diabetes (coef: 0.09; *p*: 0.083) (Table 5), consistent with the hypothesis that betel quid use decreased men's intake of bazaar‐sourced foods and thus their diabetes risk. The path through food from the bazaar did not fully explain the association between betel quid use and diabetes among men. SEM among women identified only a weak effect suggesting a potential path through food sourced from the bazaar (Table 5).

**TABLE 5:** Candidate paths between betel quid use and diabetes estimated via structural equation models controlling for pertinent confounders.*

Betel‐ > mediator			Mediator‐ > diabetes		
Coef	*P*	Mediator	Coef	*P*	Mediation effect Y/N
Men					
Direct Path: −0.15 (*p* = 0.005)					
−0.02	0.656	BMI Direct Path: (−0.14; *p* = 0.006)	0.19	< 0.001	N
−0.01	0.805	Inflammation (CRP > 3‐ < 10 mg/L) Direct Path: (−0.15; *p* = 0.007)	−0.03	0.535	N
0.06	0.209	Grip Strength Direct Path: (−0.15; *p* = 0.004)	−0.09	0.142	N
0.03	0.528	Food security Direct Path: (−0.15; *P* = 0.004)	0.08	0.103	N
−0.15	0.003	Food from the bazaar Direct Path: (−0.14; *p* = 0.012)	0.09	0.083	N
0.02	0.744	Social Network Size Direct Path: (−0.15; *P* = 0.005)	0.01	0.923	N
Women					
Direct Path: −0.03 (*p* = 0.535)					
−0.05	0.244	BMI Direct Path: (−0.01; *p* = 0.793)	0.17	< 0.001	N
−0.07	0.135	Inflammation (CRP > 3‐ < 10 mg/l) Direct Path: (−0.00; *p* = 0.922)	0.16	< 0.001	N
0.02	0.578	Grip Strength Direct Path: (−0.03; *p* = 0.501)	−0.02	0.624	N
0.02	0.626	Food security Direct Path: (−0.03; *p* = 0.503)	0.07	0.079	N
−0.15	< 0.001	Food from the bazaar Direct Path: (−0.02; *p* = 0.587)	0.02	0.546	N
0.13	0.004	Social Network Size Direct Path: (−0.03; *p* = 0.499)	0.06	0.131	N

* These models controlled for age, MacArthur Ladder, education, and laborer for men and age, MacArthur Ladder, and education for women.

## 4 Discussion

Our analysis of a large population‐based sample from rural Bangladesh suggests that use of betel quid is associated with lower risk for diabetes among men and possibly women. This relationship persisted after a thorough assessment of confounding and mediation. Our analyses suggest these associations are due to direct pathways between betel quid use and diabetes, as well as a potential indirect pathway through lower consumption of bazaar‐sourced foods among men. Weak results for women for the same mediator also suggest that more frequent use of betel quid among women might show a similar trend to men.

The gender difference we observed in associations between betel quid use and diabetes has several possible explanations. One is statistical: men are generally at higher risk for T2D compared with women and, in our data, used betel quid more heavily than women; we are thus in a better position to detect the effects of betel use at higher intensity in men (Kautzky‐Willer et al. 2023). In addition, men are far more likely to be labor migrants. If labor migration is less likely among both betel users and those with diabetes (i.e., these are two independent causes of non‐migration), then restricting our sample to current residents of Matlab could have created a spurious inverse association between betel quid use and diabetes. However, models of the diabetes outcome among men are similar when the sample is restricted to age ≥ 55 years (the age after which long‐term labor migrants have generally returned to Matlab (Alam et al. 2017))C, so we do not think missing labor migrants explain our findings.

The gender difference we observed could also be physiological, if there is dimorphism in an aspect of physiology that is part of the pathway by which betel acts. For example, the gender difference could be due to sex hormones: testosterone (T) has opposite effects on risk for metabolic disorders among men (lower T, higher risk) and women (higher T, higher risk) (Kim and Halter 2014). Betel quid increases testosterone in mouse models; if it acts similarly on testosterone in humans, this could contribute to the gender difference in associations with diabetes that we report (Yang et al. 2004). Finally, the difference could be behavioral or cultural. In Matlab, men often do more physical labor than women, especially in families that grow or raise some or all of their own food. Betel quid might reduce diabetes risk if those who use it have higher physical activity levels (Lo et al. 2016); overall lower levels of physical activity among women in our study population may have prevented them from exhibiting the protective effect we observed among men (Lo et al. 2016). These statistical, physiological, behavioral, and cultural explanations are not mutually exclusive.

Our findings differ from studies among humans in other settings that support the hypothesis that betel quid use increases risk for T2D (Lin et al. 2008; Liu et al. 2024; Mannan et al. 2000; Tseng 2010; Tung et al. 2004; Yamada et al. 2013), but accord with some laboratory‐based studies of betel quid components in animal models (Amudhan and Begum 2008; Huang et al. 2013; Musdja et al. 2020). Tung and colleagues found a positive association between betel quid use and T2D in a large, population‐based study of Taiwanese men with similar controls for confounding as our models, and a dose–response relationship between frequency of use and T2D, which supports causal inference (Tung et al. 2004). Mannan and colleagues found a positive association between betel quid use and diabetes among Bangladeshi women, and a slight decrease in glucose concentration among Bangladeshi men living in East London (Mannan et al. 2000). Among patients with diagnosed diabetes in Bangladesh, Marzan and colleagues found higher random blood glucose among betel quid users than non‐users (Al Marzan et al. 2024).

It is difficult to say whether these studies and our findings, taken together, should be seen as contradictory, given the small number of existing studies and the complexity of the potential pathways between betel quid use and T2D. To date, associations between betel quid use and diabetes have been assessed in Taiwan, Papua New Guinea, UK (among Bangladeshi migrants), and now Bangladesh—widely varying social and environmental contexts. It is possible that some component(s) of this context is important to the impact of betel quid use on diabetes risk. Potentially important differences include the way betel quid is typically prepared, and men's and women's social roles, diets, frequency of betel quid use, and levels of physical activity. Laboratory studies have identified pathways between betel quid components and metabolic processes that might both increase and decrease diabetes risk (Table 1) (Ahmed et al. 2022; Amudhan and Begum 2008; Cardosa et al. 2021; Hsieh et al. 2011; Huang et al. 2013; Musdja et al. 2020; Owen et al. 2008; Santhakumari et al. 2006). It is thus possible that the net effect of betel quid use increases diabetes risk in one setting and decreases it in another. For this reason, it is crucial for future research to assess how these components, such as tannins, flavonoids, and triterpenoids, which can have multiple, complex impacts on disease risk (e.g., anti‐inflammatory, anti‐microbial, and cholesterol‐lowering effects), interact with diets, physical activity, and other factors to influence metabolism and diabetes risk (Jing et al. 2022; Sohag et al. 2022).

Our finding of an inverse association between betel quid use and diabetes in Matlab was unexpected based on the epidemiologic literature. We thus thoroughly interrogated this association via control for a wide range of hypothesized confounders and mediators. Since no confounding or mediating variables fully explained the observed association, it is conceivable that betel quid use protects against disordered metabolism and diabetes in this population. These findings may be best understood through an evolutionary perspective—considering the potential benefits that non‐nutritive substances may have had in pre‐industrial contexts, and the way that individuals may trade off potential harms with potential benefits, particularly in contexts with limited access to biomedical health care (Perez et al. 2025).

We may not be the first to document an inverse association between diabetes and betel quid use. A study has been cited in the popular press as documenting a protective effect of betel quid against diabetes in Papua New Guinea (The Syndey Morning Herald Reporter 2008), though we were unable to locate any peer‐reviewed publications or conference proceedings to substantiate this reporting. While it may be appropriate that this study was not published (e.g., if review identified significant flaws), we are nonetheless concerned that publication bias, in which null findings or those that are opposite to predictions are less likely to be submitted or accepted for publication, may limit the literature on this topic (Laitin et al. 2021; Rosenthal 1979). This could inhibit understanding of whether, when, and how betel quid components affect metabolism and T2D risk.

### 4.1 Limitations

The cross‐sectional nature of our study design introduces some limitations. Most notably, we sampled prevalent, rather than incident, cases of diabetes and so may have oversampled cases with long survival times. It is thus possible that the inverse association we observed between betel quid use and diabetes reflects shorter survival of diabetic people who chew betel quid, rather than lower risk for diabetes among people who use betel quid. In addition, reverse causation is possible, such that diabetes causes people to chew less betel quid (rather than betel use reducing diabetes risk). While we cannot dismiss either of these possibilities, the magnitude and consistency of the association we observed among participants suggest neither alone is an adequate explanation.

We found evidence of mediation among women, but were unable to identify a pathway in SEM. This suggests that, despite having examined six potential mediators, there is some aspect of this effect we have been unable to capture. We anticipate that future research may be able to better unpack this relationship.

In this study, we were unable to distinguish between T2D and Type 1 diabetes (T1D). However, 90%–95% of diabetes diagnoses in Bangladesh are T2D, and T1D might often have been fatal in this region until treatment became available in the recent past (Biswas et al. 2019). In addition, no one in the sample reported having T1D or being on insulin. We thus have reason to believe that very few, if any, cases of T1D are included in our sample. Further, our analysis did not consider participants' report of past diagnosis of diabetes, in favor of HbA_1c_. Results might have differed if past diagnoses were taken into consideration, but this would not have allowed for stratified analysis of the HbA_1c_ result, and reported diagnoses were fairly rare. In addition, we relied on a point of care test to estimate HbA_1c_, rather than a clinical laboratory method, which could have introduced some non‐differential error.

Our sample of men may be particularly unhealthy due to the “healthy migrant effect” in which healthier men are more likely to work as labor migrants (Alam et al. 2024; Lu 2008). This introduces the possibility that both betel quid use and diabetes among men are negatively associated with migrating and, as a result, spuriously inversely associated in a sample restricted to non‐migrants. However, our findings hold in models restricted to men 55 years of age or older, a sample that includes large numbers of returned labor migrants and has likely excluded few labor migrants (Alam et al. 2017), suggesting they were not a product of our sampling strategy alone.

Further, it is possible that residual confounding by diet, physical activity, comorbidities, or other unmeasured risk factors could be influencing the associations we observed. Our sensitivity analysis using E‐values indicates that unmeasured confounding would need to have a moderate to strong relationship with both the exposure and outcome, with a risk ratio equivalent to the E‐value, with the lower bound of 1.31 for the dichotomous diabetes outcome and of 1.19 for the ordinal diabetes outcome to fully explain away the observed associations. While it is always possible to have unmeasured confounders in observational research, we considered many important covariates (age, education, MacArthur Scale of Socioeconomic Status, occupation as a proxy for physical activity among men), and potential mediation by food security, food source, BMI, CRP, grip strength, and social network size, and are confident they do not explain our results.

We also do not have data on exactly how betel quid was prepared, nor if preparation differed by gender or participants' duration of use/age at first use. This limits our ability to explain why our findings differ from other publications.

We relied on self‐reports and so our findings are vulnerable to bias and error in reporting. In particular, participants may have responded to survey items in ways they anticipated would be positively received by the interviewer (e.g., under‐reporting betel quid use); biased error or misreporting by diabetes status could have contributed to our observed associations. However, we have no reason to suspect that participants' recall differed between diabetic and non‐diabetic participants.

## 5 Conclusion

We found an inverse association between betel quid use and diabetes among men in Matlab, Bangladesh that is consistent with the hypothesis that betel quid use might protect against diabetes in this context. While our study has some limitations that should be considered when interpreting the results, in light of the contrast between laboratory‐based and epidemiologic studies, the inconsistent findings across populations, and the possibility of publication bias, this finding suggests that it is time to more closely examine the effects of betel quid components on metabolism and diabetes risk. A better understanding of the effects of betel quid on metabolic disorders is relevant to several potentially fruitful future research directions, including developing diabetes therapeutics, better elucidating diabetes pathobiology, and better understanding the widespread use of non‐nutritive substances.

## Author Contributions

**Kristin K. Sznajder:** conceptualization, data curation, formal analysis, writing – original draft preparation; **Mary K. Shenk:** conceptualization, data curation, funding acquisition, methodology, project administration, supervision, writing – review and editing; **Laura Perez:** data curation, writing – review and editing; **Nurul Alam:** methodology, project administration, resources, supervision, writing – review and editing; **Rubhana Raqib:** project administration, resources, supervision, writing – review and editing; **Anjan Kumar:** investigation, writing – review and editing; **Farjana Haque:** investigation, writing – review and editing; **Tami Blumenfield:** funding acquisition, writing – review and editing; **Siobhán M. Cully:** funding acquisition, writing – review and editing; **Katherine Wander:** conceptualization, data curation, methodology, project administration, supervision, writing – review and editing.

## Funding

This work was supported by the U.S. National Science Foundation, BCS‐1839269 and by the Pennsylvania State University.

## Conflicts of Interest

The authors declare no conflicts of interest.

## Supporting information


**Table S1:** Health outcomes that have been associated with betel quid use.
**Table S2:** Variables of interest by betel quid use (*N* = 1127).
**Table S3:** Bivariate associations between diabetes and variables of interest among men n = 410.
**Table S4:** Bivariate associations between diabetes and variables of interest among women n = 717.
**Table S5:** Regression models of the interaction between betel quid use and sex in the association with diabetes.
**Table S6:** Crude and adjusted logistic regression models of the association between betel quid use and diabetes among men ≥ 55 years of age.

## Data Availability

The data that support the findings of this study are openly available in Scholarsphere at https://scholarsphere.psu.edu/, reference number doi:10.26207/jg9r‐pe85.
