# Supplementary material for: Inverse Association Between Betel Quid Use and Diabetes in Rural Bangladesh
Source: Am J Hum Biol. 2026 Feb 13;38(2):e70203. doi: 10.1002/ajhb.70203 (PMC12903070; doi:10.1002/ajhb.70203)
Supplement: Supplementary file 1 — Table S1: Health outcomes that have been associated with betel quid use. Table S2: Variables of interest by betel quid use (N = 1127). Table S3: Bivariate associations between diabetes and variables of interest among men n = 410. Table S4: Bivariate associations between diabetes and variables of interest among women n = 717. Table S5: Regression models of the interaction between betel quid use and sex in the association with diabetes. Table S6: Crude and adjusted logistic regression models of the association between betel quid use and diabetes among men ≥ 55 years of age. [file AJHB-38-e70203-s001.docx]

**Inverse association between betel quid use and diabetes in rural Bangladesh**

**Supplemental Information**

Kristin K. Sznajder^1^*, Mary K. Shenk^2^*, Laura Perez^1,2^, Nurul Alam^3^, Rubhana Raqib^3^, Anjan Kumar^3^, Farjana Haque^3^, Tami Blumenfield^4,5^, Siobhán M. Cully^5^, and Katherine Wander^6^*

1. Pennsylvania State University, College of Medicine, Department of Public Health Sciences, Hershey, Pennsylvania, United States of America

2. Pennsylvania State University, Department of Anthropology, University Park, Pennsylvania, United States of America

3. icddr,b, Dhaka, Bangladesh

4. Yunnan University, School of Ethnology and Sociology, Kunming, China

5. University of New Mexico, Department of Anthropology, Albuquerque, New Mexico, United States of America

6. Binghamton University, State University of New York, Department of Anthropology, Binghamton, New York, United States of America

* kek157@psu.edu (KKS); mks74@psu.edu (MKS); katherinewander@binghamton.edu (KW)

| **Table S1.** Health outcomes that have been associated with betel quid use. | | | |
| --- | --- | --- | --- |
| **Health domain** | **Outcome** | **Effect of betel quid** | **Potential mechanism** |
| **Oral health** | Periodontal disease | Betel quid and its components have been associated with periodontal disease in several South Asian populations (Berniyanti, Jamaludin, Eky, Bramantoro, & Palupi, 2024; Park, Pitchumani, & Tatakis, 2024). | Arecoline has been shown to affect cellular function of the periodontium while other cholinergic compounds increase salivation and calcium deposits on teeth contributing to plaque formation and subsequent periodontal disease (Anand et al., 2014; Y.-J. Chen et al., 2015). Investigations of oral microbes in betel quid chewers found evidence of pathogens associated with periodontitis. |
|  | Precancerous lesions: oral submucous fibrosis, leukoplakia | Among studies investigating the effects of betel quid on oral health, chronic betel quid use has been associated with oral submucous fibrosis and leukoplakia, two pre-cancerous lesions (C. Lee et al., 2003; Thomas et al., 2008). One study in Myanmar found that addition of chewing tobacco to betel quid preparations significantly increased the risk of malignant lesions (Zaw et al., 2016). | Development of oral lesions has been associated with the mechanical irritation induced by betel quid placement as well as the effects of its metabolites on local cellular processes(M. Gupta, Mhaske, & Ragavendra, 2008; Prabhu et al., 2014). |
|  | Head and neck cancers (e.g., squamous cell carcinoma of the oropharynx) | All available studies have situated betel quid chewing as a major risk factor for oropharyngeal cancer (and other types of head and neck cancer) with studies in India and Taiwan attributing more than 40% of oral cancers to betel quid chewing (Guha, Warnakulasuriya, Vlaanderen, & Straif, 2014; B. Gupta & Johnson, 2014; P. Gupta & Warnakulasuriya, 2002). | The progression of pre-cancerous lesions among betel chewers underlies the elevated rates of oral cancers in South Asia (Warnakulasuriya & Chen, 2022). Oral microbiome alterations due to betel quid chewing result in colonization of pathogenic microbes that increase the risk of both precancerous lesions and head and neck cancers. |
| **Cardiovascular system** | Coronary artery disease (CAD) | Betel quid chewers have been found to be at an increased risk for coronary artery disease when compared to non-chewers (M. S. Khan et al., 2013; Mumtaz, Goyal, & Dwivedi, 2019; Tsai et al., 2012). | Metabolites from betel quid are thought to inflammation, damage to the vascular endothelium, and increase circulating lipids contributing to the development of coronary artery disease (IARC Working Group on the Evaluation of Carcinogenic Risks to Humans, 2004). |
|  | Atherosclerosis | Habitual betel quid chewing has been associated with an increased risk of atherosclerosis among both active betel chewers and ex-betel chewers (Itaki & Taufa, 2024; McClintock et al., 2014; Wei et al., 2017). | Like coronary disease, the atherosclerotic process is driven by chronic inflammation resulting from betel quid metabolites (IARC Working Group on the Evaluation of Carcinogenic Risks to Humans, 2004; Itaki & Taufa, 2024; McClintock et al., 2014; Wei et al., 2017). |
|  | Hypertension | Betel quid chewing has been associated with an increased risk of elevated blood pressure among most studies (Heck et al., 2012; Tseng, 2008). However, one study found an inverse relationship between betel quid use and blood pressure attributed to gene-by-environment interactions (Chung et al., 2007). | Betel quid metabolites have sympathetic effects which increase heart rate and vasoconstriction contributing to elevated blood pressure (Itaki & Taufa, 2024). Betel quid chewers with a genetic variation at the angiotensin enzyme converting gene have decreased risk of hypertension compared to non-chewers with the same variant (Chung et al., 2007). |
| **Reproductive health** | Preterm labor & low birth weight | Studies have found an inconsistent relationship between betel quid use and preterm labor and low birth weight (De Silva et al., 2019; Islam et al., 2024; M.-S. Yang et al., 2008). | Betel quid metabolites’ effects on the placenta and fetus are thought to be similar to nicotine’s effect, reducing placental blood flow and affecting maternal nutrition (Senn et al., 2009). |
| **Gastrointestinal & metabolic** | Liver toxicity | The long-term hepatotoxic effects of betel quid are not consistent, but some studies have found that an increased risk of liver disease in individuals with pre-existing conditions (e.g., metabolic syndrome, non-alcoholic fatty liver disease) (Chou et al., 2021; Chou et al., 2022; Singroha & Kamath, 2016). | In vitro studies suggest that betel nut alkaloids induce reactive oxygen species in hepatocytes and fibroblast activity (Hsiao, Liao, Hsieh, & Wong, 2007). |
|  | Type 2 diabetes | Most epidemiologic studies have identified a positive association between betel quid chewing and risk of type 2 diabetes (Y.-C. Huang et al., 2022; Mannan et al., 2000; Tseng, 2010; Tung et al., 2004). However, this contrasts with laboratory studies which have found anti-diabetic effects of metabolites from betel quid components (Ahmed et al., 2022; Amudhan & Begum, 2008; Arambewela et al., 2005; P.-L. Huang et al., 2013; Musdja et al., 2020; Santhakumari et al., 2006). | Betel quid may increase the risk of diabetes by increasing circulating blood glucose through its effect on glucagon secretion pathways, direct toxic effects on pancreatic beta cells, or increased central adiposity(IARC Working Group on the Evaluation of Carcinogenic Risks to Humans, 2004). Alternatively, laboratory studies suggest that betel metabolites may reduce intestinal glucose absorption, alter glucose metabolism, or have insulin-like activity (Amudhan & Begum, 2008; Santhakumari et al., 2006; Sun, Yu, Li, Hu, & Wang, 2024). |
|  | Metabolic syndrome | Betel quid chewing has been associated with metabolic syndrome and its component parts (e.g., abdominal obesity, cholesterol) (Aung, Zin, Ko, & Thet, 2023; Y.-C. Huang et al., 2022; Yamada et al., 2013). | Betel quid metabolites have been implicated in fat cell dysfunction resulting in dyslipidemia (Hsu et al., 2010). |
| **Neuropsychiatric** | Schizophrenia | An early study in 2000 and a follow-up in 2007 found schizophrenic patients that chewed betel quid had milder symptoms than those than patients who were non-chewers (Sullivan et al., 2000; Sullivan et al., 2007) | Betel quid metabolites have muscarinic activity resulting inducing parasympathetic effects and possibly the reduction of psychotic symptoms (Sullivan et al., 2000). |
| **Anemia** | Anemia | Betel quid has been associated with increased risk for anemia among men and both non-pregnant and pregnant women (Sawiah, Retnaningtyas, Siwi, & Wulandari, 2024; K. K. Sznajder et al., 2023). | Betel quid consumption may cause anemia through mechanical damage in the oral cavity which may result in inflammation or blood loss, or through damage in the gastrointestinal tract (Faouzi, Neupane, Yang, Williams, & Penner, 2018; IARC Working Group on the Evaluation of Carcinogenic Risks to Humans, 2004; Jeng et al., 2002). |

| **Table S2. Variables of interest by betel quid use (N=1127)** | | | | |
| --- | --- | --- | --- | --- |
|  | **N (column %)/ Mean (SD)** | | | |
|  | Total | Betel quid use n=599 (53.1%) | No Betel quid use n=528 (46.9%) | *p*-value |
| Diabetes  Prediabetes  No Diabetes | 175 (15.5)  460 (40.8)  492 (43.7) | 91 (15.2)  246 (41.1)  262 (43.7) | 84 (15.9)  214 (40.5)  230 (43.6) | 0.944 |
| Men  Women | 410 (36.4)  717 (63.6) | 216 (36.1)  383 (63.9) | 194 (36.7)  334 (63.3) | 0.812 |
| Age | 52.2 (+12.6) | 57.4 (+10.2) | 46.3 (+12.4) | <0.001 |
| MacArthur Ladder | 4.3 (+1.7) | 4.3 (+1.7) | 4.3 (+1.7) | 0.864 |
| Education | 4.4 (+4.2) | 2.9 (+3.5) | 6.0 (+4.3) | <0.001 |
| No school  Up to Primary  More than Primary | 355 (31.8)  385 (34.4)  378 (33.8) | 259 (43.6)  221 (37.2)  114 (19.2) | 96 (18.4)  164 (31.3)  264 (50.4) | <0.001 |
| Food secure  Not food secure | 752 (67.0)  370 (33.0) | 395 (66.1)  203 (33.9) | 357 (68.1)  167 (31.9) | 0.461 |
| Food from the bazaar  Not all food from bazaar | 646 (57.5)  477 (42.5) | 309 (51.8)  288 (48.2) | 337 (64.1)  189 (35.9) | <0.001 |
| Men who are laborers  Men of other occupation | 218 (54.4)  183 (45.6) | 128 (60.7)  83 (39.3) | 90 (47.4)  100 (52.6) | 0.008 |
| BMI | 22.8 (+4.1) | 22.2 (+4.0) | 23.4 (+4.1) | <0.001 |
| Obese >27.5  Overweight 23–<27.5  Lean 18.5–<23  Underweight<18.5 | 150 (13.4)  345 (30.7)  460 (40.9)  169 (15.0) | 64 (10.7)  159 (26.6)  266 (44.5)  109 (18.2) | 86 (16.4)  186 (35.4)  194 (36.9)  60 (11.4) | <0.001 |
| CRP >3-<10  CRP <=3 | 240 (22.1)  847 (77.9) | 114 (19.6)  467 (80.4) | 126 (24.9)  380 (75.1) | 0.036 |
| Grip Strength (R1) | 20.1 (+8.8) | 19.0 (+7.3) | 21.2 (+10.2) | <0.001 |
| Social Network Size | 11.2 (+4.0) | 11.2 (+4.0) | 11.3 (+3.9) | 0.754 |

| **Table S3. Bivariate associations between diabetes and variables of interest among men n=410** | | | | |
| --- | --- | --- | --- | --- |
|  | **N (%)/ Mean (SD)** | | | |
|  | Total | Diabetes  n=82 (20.0%) | No Diabetes n=328 (80.0%) | *p*-value |
| Betel quid use  No Betel quid use | 216 (52.7)  194 (47.3) | 32 (39.0)  50 (61.0) | 184 (56.1)  144 (43.9) | 0.006 |
| Betel quid use >=5 times per day  Betel quid use <5 times per day  No Betel quid use | 135 (33.2)  78 (19.2)  194 (47.7) | 19 (23.5)  12 (14.8)  50 (61.7) | 116 (35.6)  66 (20.3)  144 (44.2) | 0.018 |
| Age | 56.2 (+12.1) | 57.6 (+11.5) | 55.9 (+12.2) | 0.268 |
| MacArthur Ladder | 3.9 (+1.2) | 4.3 (+1.2) | 3.8 (+1.2) | <0.001 |
| Education | 4.9 (+4.5) | 5.7 (+5.0) | 4.7 (+4.3) | 0.050 |
| No school  Up to Primary  More than Primary | 113 (27.8)  137 (33.7)  156 (38.4) | 21 (25.6)  23 (28.1)  38 (46.3) | 92 (28.4)  114 (35.2)  118 (36.4) | 0.241 |
| Food secure  Not food secure | 280 (68.6)  128 (31.4) | 67 (81.7)  15 (18.3) | 213 (65.3)  113 (34.7) | 0.004 |
| Food from the bazaar  Not all food from bazaar | 194 (47.7)  213 (52.3) | 50 (61.0)  32 (39.0) | 144 (44.3)  181 (55.7) | 0.007 |
| Laborer  Other occupation | 218 (54.4)  183 (45.6) | 28 (34.6)  53 (65.4) | 190 (59.4)  130 (40.6) | <0.001 |
| BMI | 21.5 (3.6) | 23.2 (+4.4) | 21.0 (+3.3) | <0.001 |
| Obese >27.5  Overweight 23–<27.5  Lean 18.5–<23  Underweight<18.5 | 29 (7.1)  100 (24.5)  188 (45.9)  92 (22.5) | 17 (20.7)  27 (32.9)  20 (24.4)  18 (21.9) | 12 (3.7)  73 (22.3)  168 (51.4)  74 (22.6) | <0.001 |
| CRP >3-<10  CRP <=3 | 78 (19.8)  317 (80.3) | 15 (19.5)  62 (80.5) | 63 (19.8)  255 (80.2) | 0.948 |
| Grip Strength (R1) | 26.4 (+10.7) | 24.7 (+7.9) | 26.8 (+11.3) | 0.116 |
| Social Network Size | 12.7 (+3.9) | 12.8 (+4.5) | 12.7 (+3.9) | 0.883 |

| **Table S4. Bivariate associations between diabetes and variables of interest among women n=717** | | | | |
| --- | --- | --- | --- | --- |
|  | **N (%)/ Mean (SD)** | | | |
|  | Total | Diabetes n=93  (13.0%) | No Diabetes n=624 (87.0%) | *p*-value |
| Betel quid use  No Betel quid use | 383 (53.4)  334 (46.6) | 59 (63.4)  34 (36.6) | 324 (51.9)  300 (48.1) | 0.038 |
| Betel quid use >=5 times per day  Betel quid use <5 times per day  No Betel quid use | 159 (22.2)  223 (31.2)  334 (46.7) | 23 (24.7)  36 (38.7)  34 (36.6) | 136 (21.8)  187 (30.0)  300 (48.2) | 0.100 |
| Age | 49.9 (+12.3) | 55.6 (+11.2) | 49.1 (+12.3) | <0.001 |
| MacArthur Ladder | 4.6 (+1.8) | 5.0 (+1.8) | 4.5 (+1.8) | 0.011 |
| Education | 4.1 (+3.9) | 3.3 + (3.8) | 4.2 (+3.9) | 0.030 |
| No school  Up to Primary  More than Primary | 242 (33.9)  248 (34.8)  222 (31.2) | 41 (44.6)  33 (35.9)  18 (19.6) | 201 (32.4)  215 (34.7)  204 (32.9) | 0.018 |
| Food secure  Not food secure | 472 (66.1)  242 (33.9) | 70 (76.1)  22 (23.9) | 402 (64.6)  220 (35.4) | 0.030 |
| Food from the bazaar  Not all food from bazaar | 452 (63.1)  264 (36.9) | 63 (67.7)  30 (32.3) | 389 (62.4)  234 (37.6) | 0.323 |
| BMI | 23.5 (+4.2) | 25.1 (+4.1) | 23.3 (+4.2) | <0.001 |
| Obese >27.5  Overweight 23–<27.5  Lean 18.5–<23  Underweight<18.5 | 121 (16.9)  245 (34.3)  272 (38.0)  77 (10.8) | 28 (30.4)  33 (35.9)  29 (31.5)  2 (2.2) | 93 (14.9)  212 (34.0)  243 (39.0)  75 (12.0) | <0.001 |
| CRP >3-<10  CRP <=3 | 162 (23.4)  530 (76.6) | 36 (41.9)  50 (58.1) | 126 (20.8)  480 (79.2) | <0.001 |
| Grip Strength (R1) | 16.4 (+4.6) | 15.7 (+4.7) | 16.6 (+4.5) | 0.086 |
| Social Network Size | 10.4 (+3.8) | 10.9 (+3.9) | 10.3 (+3.7) | 0.149 |

| **Table S5. Regression models of the interaction between betel quid use and sex in the association with diabetes** | | |  |
| --- | --- | --- | --- |
| **Interaction Term** | **Logistic regression**  **(HbA_1c_ ≥ 6.5%)** | **Ordinal logistic regression**  **(HbA_1c_ < 5.6%; 5.7% ≤ HbA_1c_ < 6.5; HbA_1c_ ≥ 6.5%)** | |
|  | **Regression Coefficient (p-value)** | **Regression Coefficient (p-value)** | |
| Betel quid use | 0.5 (0.039) | 0.2 (0.812) | |
| Male Sex | 1.1 (<0.001) | 0.2 (0.129) | |
| Betel quid use * Sex | -1.2 (<0.001 | -0.7 (0.005) | |

| **Table S6. Crude and adjusted logistic regression models of the association between betel quid use and diabetes among men >55 years of age** | | |
| --- | --- | --- |
|  | **Logistic regression**  **(HbA_1c_ ≥ 6.5%)** | **Ordinal logistic regression**  **(HbA_1c_ < 5.6%; 5.7% ≤ HbA_1c_ < 6.5; HbA_1c_ ≥ 6.5%)** |
| **Panel 1 (Crude)** | **OR (CI)** | **OR (CI)** |
| Betel quid use | 0.29 (0.16, 0.56) | 0.45 (0.27, 0.76) |
| **Panel 2 (Adjusted for confounders)** | **OR (CI)** | **OR (CI)** |
| Betel quid use | 0.36 (0.18, 0.71) | 0.52 (0.30, 0.89) |
| Age | 0.99 (0.94, 1.04) | 0.99 (0.96, 1.03) |
| MacArthur Ladder | 1.12 (0.84, 1.49) | 1.06 (0.85, 1.32) |
| Education | 1.04 (0.96, 1.12) | 1.02 (0.96, 1.08) |
| Laborer | 0.46 (0.22, 0.98) | 0.57 (0.33, 1.01) |
| **Panel 3 (Adjusted for confounders and mediators)** | **OR (CI)** | **OR (CI)** |
| Betel quid use | 0.35 (0.17, 0.74) | 0.62 (0.35, 1.10) |
| Age | 0.99 (0.94, 1.05) | 0.99 (0.96, 1.04) |
| MacArthur Ladder | 1.19 (0.84, 1.67) | 1.05 (0.83, 1.34) |
| Education | 0.98 (0.88, 1.08) | 1.01 (0.94, 1.09) |
| Laborer | 0.67 (0.26, 1.74) | 0.72 (0.37, 1.42) |
| Food security | 1.33 (0.53, 3.36) | 1.04 (0.56, 1.92) |
| Food from the bazaar | 1.67 (0.68, 4.08) | 1.36 (0.72, 2.56) |
| BMI | 1.14 (1.02, 1.27) | 1.10 (1.02, 1.19) |
| CRP >3<10 | 0.92 (0.35, 2.39) | 0.88 (0.44, 1.75) |
| Grip strength | 0.98 (0.93, 1.04) | 0.99 (0.97, 1.02) |
| Social network size | 1.02 (0.93, 1.13) | 0.98 (0.92, 1.06) |

**References**

Ahmed, S., Ali, M. C., Ruma, R. A., Mahmud, S., Paul, G. K., Saleh, M. A., . . . Rahman, M. M. (2022). Molecular docking and dynamics simulation of natural compounds from betel leaves (Piper betle L.) for investigating the potential inhibition of alpha-amylase and alpha-glucosidase of type 2 diabetes. *Molecules, 27*(14), 4526.

Amudhan, M., & Begum, V. (2008). Alpha-glucosidase inhibitory and hypoglycemic activities of Areca catechu extract. *Pharmacognosy magazine, 4*(15), 223.

Anand, R., Dhingra, C., Prasad, S., & Menon, I. (2014). Betel nut chewing and its deleterious effects on oral cavity. *Journal of cancer research and therapeutics, 10*(3), 499.

Arambewela, L. S. R., Arawwawala, L., & Ratnasooriya, W. D. (2005). Antidiabetic activities of aqueous and ethanolic extracts of Piper betle leaves in rats. *Journal of Ethnopharmacology, 102*(2), 239-245.

Aung, A. A., Zin, S. N. S., Ko, A. K., & Thet, A. C. (2023). The Association between Betel Quid Chewing and Metabolic Syndrome Among Urban Adults in Mandalay District of Myanmar. *Journal of the ASEAN Federation of Endocrine Societies, 38*(2), 50.

Berniyanti, T., Jamaludin, M. B., Eky, Y. E., Bramantoro, T., & Palupi, R. (2024). Duration and frequency of betel quid chewing affects periodontitis severity and life quality of people in Tanini Village, Kupang, Indonesia. *International Journal of Dental Hygiene, 22*(1), 229-235.

Chen, Y.-J., Lee, S.-S., Huang, F.-M., Yu, H.-C., Tsai, C.-C., & Chang, Y.-C. (2015). Effects of arecoline on cell growth, migration, and differentiation in cementoblasts. *Journal of Dental Sciences, 10*(4), 388-393.

Chou, Y.-T., Li, C.-H., Sun, Z.-J., Shen, W.-C., Yang, Y.-C., Lu, F.-H., . . . Wu, J.-S. (2021). A positive relationship between betel nut chewing and significant liver fibrosis in NAFLD subjects, but not in non-NAFLD ones. *Nutrients, 13*(3), 914.

Chou, Y.-T., Sun, Z.-J., Shen, W.-C., Yang, Y.-C., Lu, F.-H., Chang, C.-J., . . . Wu, J.-S. (2022). Cumulative betel quid chewing and the risk of significant liver fibrosis in subjects with and without metabolic syndrome. *Frontiers in Nutrition, 9*, 765206.

Chung, F.-M., Shieh, T.-Y., Yang, Y.-H., Chang, D.-M., Shin, S.-J., Tsai, J. C.-R., . . . Lee, Y.-J. (2007). The role of angiotensin-converting enzyme gene insertion/deletion polymorphism for blood pressure regulation in areca nut chewers. *Translational research, 150*(1), 58-65.

De Silva, M., Panisi, L., Brownfoot, F. C., Lindquist, A., Walker, S. P., Tong, S., & Hastie, R. (2019). Systematic review of areca (betel nut) use and adverse pregnancy outcomes. *International Journal of Gynecology & Obstetrics, 147*(3), 292-300.

Faouzi, M., Neupane, R. P., Yang, J., Williams, P., & Penner, R. (2018). Areca nut extracts mobilize calcium and release pro-inflammatory cytokines from various immune cells. *Scientific reports, 8*(1), 1075.

Guha, N., Warnakulasuriya, S., Vlaanderen, J., & Straif, K. (2014). Betel quid chewing and the risk of oral and oropharyngeal cancers: a meta‐analysis with implications for cancer control. *International Journal of Cancer, 135*(6), 1433-1443.

Gupta, B., & Johnson, N. W. (2014). Systematic review and meta-analysis of association of smokeless tobacco and of betel quid without tobacco with incidence of oral cancer in South Asia and the Pacific. *PloS one, 9*(11), e113385.

Gupta, M., Mhaske, S., & Ragavendra, R. (2008). Oral submucous fibrosis: current concepts in etiopathogenesis.

Gupta, P., & Warnakulasuriya, S. (2002). Global epidemiology of areca nut usage. *Addiction biology, 7*(1), 77-83.

Heck, J. E., Marcotte, E. L., Argos, M., Parvez, F., Ahmed, A., Islam, T., . . . Chen, Y. (2012). Betel quid chewing in rural Bangladesh: prevalence, predictors and relationship to blood pressure. *International journal of epidemiology, 41*(2), 462-471.

Hsiao, T.-J., Liao, H.-W. C., Hsieh, P.-S., & Wong, R.-H. (2007). Risk of betel quid chewing on the development of liver cirrhosis: a community-based case-control study. *Annals of Epidemiology, 17*(6), 479-485.

Hsu, H.-F., Tsou, T.-C., Chao, H.-R., Shy, C.-G., Kuo, Y.-T., Tsai, F.-Y., . . . Ko, Y.-C. (2010). Effects of arecoline on adipogenesis, lipolysis, and glucose uptake of adipocytes—A possible role of betel-quid chewing in metabolic syndrome. *Toxicology and applied pharmacology, 245*(3), 370-377.

Huang, P.-L., Chi, C.-W., & Liu, T.-Y. (2013). Areca nut procyanidins ameliorate streptozocin-induced hyperglycemia by regulating gluconeogenesis. *Food and chemical toxicology, 55*, 137-143.

Huang, Y.-C., Geng, J.-H., Wu, P.-Y., Huang, J.-C., Chen, S.-C., Chang, J.-M., & Chen, H.-C. (2022). Betel nut chewing increases the risk of metabolic syndrome and its components in a large Taiwanese population follow-up study category: original investigation. *Nutrients, 14*(5), 1018.

IARC Working Group on the Evaluation of Carcinogenic Risks to Humans. (2004). Betel-quid and areca-nut chewing and some areca-nut derived nitrosamines. *IARC Monogr Eval Carcinog Risks Hum, 85*(1).

Islam, M. R., Aktar, S., Pervin, J., Rahman, S. M., Rahman, M., Rahman, A., & Ekström, E.-C. (2024). Maternal betel quid use during pregnancy and child growth: a cohort study from rural Bangladesh. *Global health action, 17*(1), 2375829.

Itaki, R., & Taufa, S. (2024). Association between habitual betel quid chewing and risk of adverse cardiovascular outcomes: A systematic review. *Tropical Medicine & International Health*.

Jeng, J.-H., Chen, S.-Y., Liao, C.-H., Tung, Y.-Y., Lin, B.-R., Hahn, L.-J., & Chang, M.-C. (2002). Modulation of platelet aggregation by areca nut and betel leaf ingredients: roles of reactive oxygen species and cyclooxygenase. *Free Radical Biology and Medicine, 32*(9), 860-871.

Khan, M. S., Bawany, F. I., Ahmed, M. U., Hussain, M., Khan, A., & Lashari, M. N. (2013). Betel nut usage is a major risk factor for coronary artery disease. *Global journal of health science, 6*(2), 189.

Lee, C., Ko, Y., Huang, H., Chao, Y., Tsai, C., Shieh, T., & Lin, L. (2003). The precancer risk of betel quid chewing, tobacco use and alcohol consumption in oral leukoplakia and oral submucous fibrosis in southern Taiwan. *British journal of cancer, 88*(3), 366-372.

McClintock, T. R., Parvez, F., Wu, F., Wang, W., Islam, T., Ahmed, A., . . . Desvarieux, M. (2014). Association between betel quid chewing and carotid intima-media thickness in rural Bangladesh. *International journal of epidemiology, 43*(4), 1174-1182.

Mumtaz, S. M., Goyal, R. K., & Dwivedi, S. (2019). Adverse cardiovascular effects of betel nut. *MGM Journal of Medical Sciences, 6*(4), 171-174.

Musdja, M. Y., Nurdin, A., & Musir, A. (2020). *Antidiabetic effect and glucose tolerance of areca nut (Areca catechu) seed ethanol extract on alloxan-induced diabetic male rats.* Paper presented at the IOP Conference Series: Earth and Environmental Science.

Park, J. V., Pitchumani, P. K., & Tatakis, D. N. (2024). Periodontitis presenting among betel quid users: A case series. *Clinical Advances in Periodontics*.

Prabhu, R. V., Prabhu, V., Chatra, L., Shenai, P., Suvarna, N., & Dandekeri, S. (2014). Areca nut and its role in oral submucous fibrosis. *Journal of clinical and experimental dentistry, 6*(5), e569.

Santhakumari, P., Prakasam, A., & Pugalendi, K. V. (2006). Antihyperglycemic activity of Piper betle leaf on streptozotocin-induced diabetic rats. *Journal of medicinal food, 9*(1), 108-112.

Sawiah, S., Retnaningtyas, E., Siwi, R. P. Y., & Wulandari, A. (2024). Behavior Of Consuming Betel Nut On The Incidence Anemia In Pregnant Women At Posyandu Wasur Kampung, Working Area Of Rimba Jaya Health Center Merauke District-Merauke Regency. *Journal of Health Science Community, 5*(2), 127-133.

Senn, M., Baiwog, F., Winmai, J., Mueller, I., Rogerson, S., & Senn, N. (2009). Betel nut chewing during pregnancy, Madang province, Papua New Guinea. *Drug and alcohol dependence, 105*(1-2), 126-131.

Singroha, K., & Kamath, V. V. (2016). Liver function tests as a measure of hepatotoxicity in areca nut chewers. *Journal of Dental Research and Review, 3*(2), 60-64.

Sullivan, R. J., Allen, J. S., Otto, C., Tiobech, J., & Nero, K. (2000). Effects of chewing betel nut (Areca catechu) on the symptoms of people with schizophrenia in Palau, Micronesia. *The British journal of psychiatry, 177*(2), 174-178.

Sullivan, R. J., Andres D Ch MS, P. D. M., Sylvia, Otto, C., Miles, W., & Kydd, R. (2007). The effects of an indigenous muscarinic drug, Betel nut (Areca catechu), on the symptoms of schizophrenia: a longitudinal study in Palau, Micronesia. *American Journal of Psychiatry, 164*(4), 670-673.

Sun, H., Yu, W., Li, H., Hu, X., & Wang, X. (2024). Bioactive components of areca nut: an overview of their positive impacts targeting different organs. *Nutrients, 16*(5), 695.

Sznajder, K. K., Shenk, M. K., Alam, N., Raqib, R., Kumar, A., Haque, F., . . . Wander, K. (2023). Betel quid use is associated with anemia among both men and women in Matlab, Bangladesh. *PLOS Global Public Health, 3*(6), e0001677.

Thomas, S. J., Harris, R., Ness, A. R., Taulo, J., Maclennan, R., Howes, N., & Bain, C. J. (2008). Betel quid not containing tobacco and oral leukoplakia: a report on a cross‐sectional study in Papua New Guinea and a meta‐analysis of current evidence. *International Journal of Cancer, 123*(8), 1871-1876.

Tsai, W.-C., Wu, M.-T., Wang, G.-J., Lee, K.-T., Lee, C.-H., Lu, Y.-H., . . . Lin, T.-H. (2012). Chewing areca nut increases the risk of coronary artery disease in Taiwanese men: a case-control study. *BMC public health, 12*, 1-7.

Tseng, C.-H. (2008). Betel nut chewing is associated with hypertension in Taiwanese type 2 diabetic patients. *Hypertension Research, 31*(3), 417-423.

Warnakulasuriya, S., & Chen, T. (2022). Areca nut and oral cancer: evidence from studies conducted in humans. *Journal of Dental Research, 101*(10), 1139-1146.

Wei, Y.-T., Chou, Y.-T., Yang, Y.-C., Chou, C.-Y., Lu, F.-H., Chang, C.-J., & Wu, J.-S. (2017). Betel nut chewing associated with increased risk of arterial stiffness. *Drug and alcohol dependence, 180*, 1-6.

Yamada, T., Hara, K., & Kadowaki, T. (2013). Chewing betel quid and the risk of metabolic disease, cardiovascular disease, and all-cause mortality: a meta-analysis. *PloS one, 8*(8), e70679.

Yang, M.-S., Lee, C.-H., Chang, S.-J., Chung, T.-C., Tsai, E.-M., Ko, A. M.-J., & Ko, Y.-C. (2008). The effect of maternal betel quid exposure during pregnancy on adverse birth outcomes among aborigines in Taiwan. *Drug and alcohol dependence, 95*(1-2), 134-139.

Zaw, K. K., Ohnmar, M., Hlaing, M. M., Oo, Y. T., Win, S. S., Htike, M. M., . . . Thein, Z. M. (2016). Betel Quid and Oral Potentially Malignant Disorders in a Periurban Township in Myanmar. *PloS one, 11*(9), e0162081. doi:10.1371/journal.pone.0162081
